# Visual acuity and contrast sensitivity are two important factors affecting vision-related quality of life in advanced age-related macular degeneration

**DOI:** 10.1371/journal.pone.0196481

**Published:** 2018-05-10

**Authors:** Miin Roh, Alexandra Selivanova, Hyun Joon Shin, Joan W. Miller, Mary Lou Jackson

**Affiliations:** 1 Department of Ophthalmology, Massachusetts Eye and Ear, Harvard Medical School, Boston, Massachusetts, United States of America; 2 Division of Aging, Brigham and Women’s Hospital, Department of Medicine, VA Boston Health care system, Harvard Medical School, Boston, Massachusetts, United States of America; 3 VGH/Univeristy of British Coumbia Eye Care Centre, Vancouver, British Columbia, Canada; Save Sight Institute, AUSTRALIA

## Abstract

**Purpose:**

Vision loss from age-related macular degeneration (AMD) has a profound effect on vision-related quality of life (VRQoL). The pupose of this study is to identify clinical factors associated with VRQoL using the Rasch- calibrated NEI VFQ-25 scales in bilateral advanced AMD patients.

**Methods:**

We retrospectively reviewed 47 patients (mean age 83.2 years) with bilateral advanced AMD. Clinical assessment included age, gender, type of AMD, high contrast visual acuity (VA), history of medical conditions, contrast sensitivity (CS), central visual field loss, report of Charles Bonnet Syndrome, current treatment for AMD and Rasch-calibrated NEI VFQ-25 visual function and socioemotional function scales. The NEI VFQ visual function scale includes items of general vision, peripheral vision, distance vision and near vision-related activity while the socioemotional function scale includes items of vision related-social functioning, role difficulties, dependency, and mental health. Multiple regression analysis (structural regression model) was performed using fixed item parameters obtained from the one-parameter item response theory model.

**Results:**

Multivariate analysis showed that high contrast VA and CS were two factors influencing VRQoL visual function scale (β = -0.25, 95% CI-0.37 to -0.12, p<0.001 and β = 0.35, 95% CI 0.25 to 0.46, p<0.001) and socioemontional functioning scale (β = -0.2, 95% CI -0.37 to -0.03, p = 0.023, and β = 0.3, 95% CI 0.18 to 0.43, p = 0.001). Central visual field loss was not assoicated with either VRQoL visual or socioemontional functioning scale (β = -0.08, 95% CI-0.28 to 0.12,p = 0.44 and β = -0.09, 95% CI -0.03 to 0.16, p = 0.50, respectively).

**Conclusion:**

In patients with vision impairment secondary to bilateral advanced AMD, high contrast VA and CS are two important factors affecting VRQoL.

## Introduction

Age-related macular degeneration (AMD) is one of the leading causes of vision loss in the elderly population which negatively impacts patients’ overall visual function and daily life.[1] There is an estimated 1.75 million people in the United States of America who have advanced AMD and an estimated 1.22 million people who have neovascular AMD in at least one eye.[2] In the 10 year follow up on patients with advanced AMD in one eye in the AREDS study [3], 26.4% of the patients developed advanced AMD in the fellow eye. Presence of large drusen or retinal pigment epithelium abnormalities increased the risk of developing advanced AMD to 61.1% within10 years in those with advanced AMD in the fellow eye. This leads to an increase in low vision within the elderly population.

As visual function declines, independence and activities of daily living are impacted with a deterioration in the overall quality of life. [4–6] Increasing numbers of patients with bilateral advanced AMD will increase health resource utilization and social economic costs.[7] This emphasizes the importance of identifying different clinical factors assoicated with reduced vision-related quality of life (VRQoL) which can be used to to identify patients in need of support and vision rehabilitation. Therefore, the primary purpose of this study was to explore which clinical features best correlate with VRQoL using the NEI-VFQ 25 questionnaire.

## Patients and methods

This was a retrospective review of patients with bilateral advanced AMD who had consented to participate in a Vision Rehabilitation registry after their consultation at the Massachusetts Eye and Ear (MEE) Vision Rehabilitation Clinic between January 2012 and September 2014. The clinical protocol was conducted in accordance with Health Insurance Portability and Accountability Act requirements, the tenets of the Declaration of Helsinki, and was approved by the Institutional Review Board at Mass. Eye and Ear. All subjects enrolled in the study provided written informed consent.

Inclusion criteria included a diagnosis of bilateral advanced AMD, completion of the NEI VFQ-25 questionnaire, and completion of scanning laser ophthalmoscopy (SLO) macular microperimetry. Advanced stage AMD was defined as having geographic atrophy (defined as sharply demarcated round/oval area of hypopigmentation with corresponding visual field scotoma or reduced sensitivities), disciform scars with subretinal fibrosis or scars, subretinal hemorrhage or visible subretinal new vessel consistent with choroidal neovascularization. Patients were excluded if they had early or intermediate AMD (defined as drusen or pigmentary changes in the macula), choroidal neovascularization caused by pathologies other than AMD, diabetic retinopathy, macular dystrophy other than AMD, advanced cataract causing severe media opacity, retinitis pigmentosa, vision problems secondary to cerebrovascular accidents, glaucoma or insufficient clinical data.

Charts of patients who met the inclusion criteria above were reviewed for demographic and clinical data including gender, age at presentation, presence of co-morbidities (cardiovascular disease, diabetes mellitus, malignancy or transient ischemic attack), prior treatment for neovascular AMD, report of visual hallucinations consistent with Charles Bonnet Syndrome (CBS), visual acuity (VA), and binocular contrast sensitivity (CS). High contrast VA was assessed in Snellen notation and converted to the logarithm of minimal angle of resolution (logMAR) scale.[8] According to the 2006 updated WHO visual impairment criteria, moderate and severe vision impairment was defined as presenting visual acuity worse than 6/18 to 3/60 and referred to as low vision.[9] In the current study, in order to assess ranges of VA, high contrast VA was categorized into 3 groups; both eyes better than 0.48 logMAR VA (equivalent to Snellen VA 20/60), one eye better than 0.48logMAR, and both eyes worse than 0.48logMAR. CS was measured binocularly at a distance of 1 meter, under controlled photopic conditions (85 cd/m^2^). The score corresponding to the last triplet of letters seen by the patient was recorded in logarithmic scale.[10]

### NEI VFQ-25 visual function scale and socioemotional scale with Rasch analysis

Patient-reported vision related quality of life (VRQoL) was assessed before the initial visit to the Vision Rehabilitation Clinic using the NEI VFQ-25 questionnaire, which measures subjective assessment of patient-reported vision-targeted health status elements that are most important to people with eye disease.[11] Rasch analysis was performed using methods suggested by Pesudovs et al.[12] and co-primary endpoints for the Rasch analysis were divided into visual functioning scale and socioemotional scale. The visual function scale was compromised of items regarding general vision (2), near activities (5, 6, 7), distance activities (8, 9, 14), and peripheral vision (10). Socioemotional function scale was comprised of vision-related social functioning (11, 13), vision-related role difficulties (17,18), vision-related dependency (20, 23, 24), and vision-related mental health (21,22,25). In these items, higher number indicated better socioemotional function. Several items (5,6,7,8,9,10,11,13,14) in the visual function scale were reversed for consistencies in the overall item polarity. Person and item measures were examined for fit to the Rasch model using infit and outfit item fit statistics.

### SLO macular microperimetry

Macular microperimetry testing was conducted with a scanning laser ophthalmoscopy (SLO) microperimetry (Optos plc, Dunfermline, Scotland). The severity of central visual field (VF) loss was assessed by having patients fixate a cross, presented in the center of the display, while a test stimulus (0.438 disk) was presented for 200 milliseconds at each of 52 radially organized locations within 20 degrees centered on the initial fixation location. All patients were tested with a suprathreshold strategy. Suprathreshold testing entails the presentation of a target at an intensity that is anticipated to be seen (0-decibel [dB] attenuation), and the patient indicates when the target is detected by pressing a button. During testing, fixation is continuously monitored and a measure of fixation stability can be obtained—defined as percentage of fixation that remained within 2 degrees and 4 degrees of initial fixation. Scotomatous (or non-responding) points were defined as testing loci that elicited no participant response at the tested intensity. Responding points were defined as all other testing loci for which a response was recorded after stimulus presentation.

**Fig 1:**
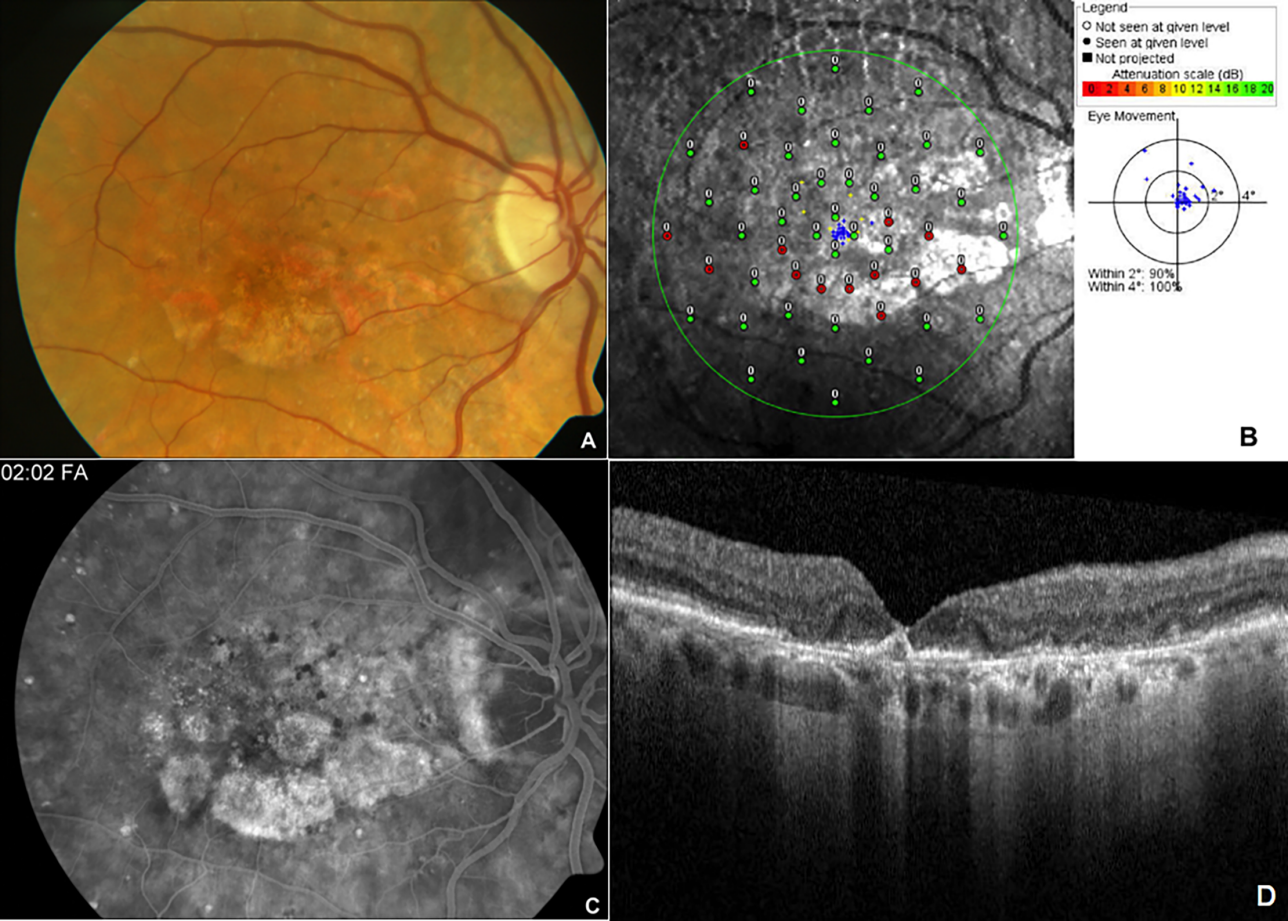
Representative color fundus photograph (A), SLO macular microperimetry (B), fluorescein angiogram (C) and optical coherence tomography (D) data of the right eye of a patient with bilateral advanced age-related macular degeneration. Color fundus photograph (A) showed patchy area of geographic atrophy with scattered drusen in the macula. SLO macular microperimetry (B) showed good fixation (blue dots) with 2–3 disc diameters sized inferior paracentral scotoma secondary to geographic atrophy sparing the central fovea as indicated by red (non-responding)/green (responding) dots leading to paracentral moderate scotoma (Score 4). Late phase angiogram (C) showed staining patterns along the atrophy area with diffuse area of outer retinal layer irregularity on OCT with geographic atrophy (D).

The configuration of geographic atrophy was classified as unifocal, multifocal, horseshoe shape, or ring shape sparing the fovea area.[13, 14] SLO macular microperimetry data of both eyes was reviewed by the senior author and a binocular summation of central VF loss was by determined by estimating the extent of overlap of scotomas from both eyes. The central field was then stratified into 13 categories based on presence, configuration, location, and size of central and paracentral scotomas (Fig 1). If the VF loss was a ring shape sparing the fovea, configuration of the central VF loss was described as ‘encircling’. Paracentral field loss was defined as scotoma that spared the fovea and central field loss was definted as scotoma that involved the fovea. The extent of central field loss was categorized according to disc diameter (DD): small if the central VF defect ≤ 1 DD; medium if the central field defect was greater than 1DD but ≤ 2DD; large if the extent of central field loss was greater than 2DD but ≤ 3DD; very large if the central VF defect was >3DD. A score of 0 to 13 was given (0 being normal and 13 being the most severe central VF loss) accordingly, and the scores were combined and categorized into one of the three following levels: mild (scores 1, 2, 3, and 6), moderate (scores 4, 5, 7, 10, 11, 12) or severe (scores 8, 9, 13).

## Statistical analysis

All data were analyzed using MINISTEP® 3.92.1 (Beaverton, OR), SAS® version 9.4 (Cary, NC) and M plus® version 7.4 (Los Angeles, CA). Continuous variables were presented as mean and standard deviation (SD), whereas categorical variables were presented as absolute (n) and relative frequency (%). VRQoL was assessed using the NEI-VFQ 25 questionnaire.[15] First, one parameter item response theory model (Rasch model) was set up to obtain final estimates of “person measures” or Rasch scores summarizing the NEI- VFQ responses regarding the two-factor model of visual functioning scale and socioemotional scale. Rasch analysis located item difficulty and person ability on a logit scale (log of odds). How well the data fits the model was evaluated by the item fit statistics infit and outfit using MINISTEP. The information-weighted (infit) statistic was more sensitive to the pattern of responses to person-targeted items and less sensitive to the presence of outliers. The outlier-sensitive (outfit) statistic was sensitive to unexpected behavior by persons or items far from the subject’s ability level. In the mean square (MNSQ) form, fit statistics showed variance in the data with an expected value of 1.0. MNSQ values less than 1.0 indicated that the items are too predictable, thereby suggesting overfitting. Values of more than 1.0 suggested unpredictability due to noise in the data and are considered to be underfitting. Infit value larger than 0.6 and outfit values less than 1.4 were considered to be a good fit in polytomous rating scale model analysis.[16] We also configured a measurement model (1 parameter item response theory graded response model) using MPLUS® to obtain the item parameters of latent traits of VRQoL. We selected a model by comparing Akaike information criterion and Bayesian information criterion with the alternative model (rating scale model).[17] Then, multiple regression analysis (structural regression model) was performed using fixed item parameters obtained from the measurement model. This method has the advantage of controlling measurement errors, therefore, providing consistent estimates of the structural regression parameters.[18] In order to find the factors associated with VRQoL with NEI-VFQ 25 scores, high contrast VA, central VF loss, CS, age, gender, history of treatment for AMD, presence of CBS and co-morbidities of diabetes mellitus, cardiovascular disease, stroke, and cancer were evaluated using multiple regression analysis. Regression coefficients were reported with 95% confidence intervals (CIs). We dealt with missing covariates using full information maximum likelihood method under the ‘missing at random assumption’. A two-tailed P value < 0.05 was considered statistically significant.

## Results

Among the 111 subjects with bilateral AMD who agreed to participate in the Vision Rehabilitation registry, 64 patients were excluded from analysis. Of the 64 patients who were excluded, 15 patients (23.8%) did not complete SLO macular microperimetry, 8 patients (12.7%) did not complete the NEI VFQ-25 questionnaire, 2 patients (3.2%) had CNV related to other causes, 3 patients (4.6%) had other ocular pathology in addition to AMD, and 36 patients (57.1%) had insufficient clinical data. In total, we identified 47 patients with advanced bilateral AMD who completed both the NEI-VFQ-25 questionnaire and SLO microperimetry.

The demographics and clinical characteristics for patients are summarized in Table 1. Fundus examination showed that 5 patients had bilateral disciform scar in the macula, 6 patients had a disciform scar in one eye and geographic atrophy in the other eye, and 36 patients had bilateral geographic atrophy.

**Table 1:** Baseline characteristics of patients with bilateral age-related macular degeneration.

AMD (n = 47)	
**Age (years, mean ± standard deviation)**	83.2 ± 6.1
**Male (%)**	18 (38.3%)
**Visual acuity (%)**	
Both eyes better than 0.48 LogMAR	4/47 (8.5%)
One eye better than 0.48 LogMAR	20/47 (42.6%)
Both eyes worse than 0.48 LogMAR	23/47 (48.9%)
**Contrast sensitivity test (Pelli-Robson test, log unit)**	1.0 ±0.3
**Charles Bonnet syndrome (%)**	13 (28%)
**Wet AMD at least in one eye (%)**	26 (55.3%)
**Current treatment for AMD at least in one eye (%)**	11 (23.4%)
**History of transient ischemic attack**	1 (2.1%)
**History of Cardiovascular disease**	38 (81%)
**History of Diabetes mellitus**	6 (13%)
**History of malignacy**	8 (17%)

AMD, age-related macular degeneration

Central VF grading analysis showed that there were 3 patients (6.3%) with near normal central VF, 26 patients (55.3%) with paracentral field loss, 12 patients (25.5%) with central field loss involving the fovea, and 6 patients (12.8%) with encircling field loss. According to the VF severity scale, 23 patients (48.9%) had mild central VF loss, 10 patients (21.3%) had moderate central VF defects, and 14 (29.8%) had severe central VF defects (Table 2).

**Table 2:** Visual field scoring system with SLO macular microperimetry in patients with bilateral age-related macular degeneration.

	Grade	Size of scotoma	Number of Patients (%)
**Normal**	0	None	0 (0)
Near normal*	1		3 (6.3)
**Paracentral field loss**			
paracentral small scotoma	2	≤ 1 DD	7 (14.6)
paracentral medium scotoma	3	> 1DD or ≤ 2DD	13 (27.1)
paracentral large scotoma	4	> 2DD and ≤ 3DD	2 (4.2)
paracentral very large scotoma	5	> 3DD	4 (8.3)
**Central Scotoma**			
central small scotoma	6	≤ 1 DD	0 (0)
central medium scotoma	7	> 1DD or ≤ 2DD	1 (2.1)
central large scotoma	8	> 2DD and ≤ 3DD	4 (8.3)
central very large scotoma	9	> 3DD	7 (14.6)
**Encircling**			
encircling small scotoma	10	≤ 1 DD	1 (2.1)
encircling medium scotoma	11	> 1DD or ≤ 2DD	1 (2.1)
encircling large scotoma	12	> 2DD and ≤ 3DD	1 (2.1)
encircling very large scotoma	13	> 3DD	3 (6.3)
**Severity of Visual field deficit**	1, 2, 3, 6,	Mild	23 (48.9)
	4, 5, 7, 10,11, 12,	Moderate	10 (21.3)
	8, 9, 13,	Severe	14 (29.8)

* scotoma detected with a target smaller than Goldmann III size

Table 3 represents the fit statistics of items subcategorized into visual functioning scale and socioemotional scale. Among the NEI-VFQ25 questionnaire, NEI item #11 (seeing how people react to things) showed disordered category threshold, therefore we combined responses category 4 (extreme difficulty) and category 5 (stopped doing this because of your eyesight) resulting in ordinal threshold. We used a minimalist approach and did not manipulate modestly under fitted (1.4<outfit<2.0) items in the visual function subscale (items #9 and #14) and the socioemotional subscale (items #23 and #25).

**Table 3:** Fit statistics of each item categorized into visual functioning subscale and socioemotional subscale of NEI VFQ-25.

	Measure	Error	Infit MNSQ	Outfit MNSQ
**Visual functioning scale**				
2. Eye sight using both eyes	-0.54	0.2	0.69	0.68
5.Reading ordinary print in news papers	2.16	0.24	1.14	0.94
7.Finding something on a crowded shelf	-0.75	0.2	0.65	0.66
6.See well up close	0.85	0.21	0.87	0.94
8.Reading street signs or names of the stores	0.28	0.2	0.69	0.68
9.Going down steps, stairs, or curbs in dim light or at night	-0.27	0.2	1.49	1.45
10. Noticing objects off to the side while you are walking along	-1.4	0.21	0.95	0.90
14.Going out to see movies, plays, or sports events	-0.34	0.21	1.61	1.54
**Socioemotional function scale**				
11. Seeing how people react to things	-0.84	0.18	1.12	1.39
13. Visiting with people in their homes, at parties, or in restaurants	-0.84	0.18	0.90	0.73
17. Do you accomplish less	0.6	0.17	0.88	0.98
18. Are you limited	-0.19	0.17	0.62	0.69
20. Stay home most of the time	-0.63	0.17	1.10	0.90
21. Frustrated	1.31	0.2	1.25	1.16
22. Much less control	0.99	0.19	0.75	0.62
24. Need a lot of help	0.41	0.17	1.16	1.14
23. Rely too much on what other people tell me	0.01	0.17	1.34	1.41
25. Doing things that will embarrass myself or others	-0.84	0.18	1.20	1.58

The Rasch analysis fit statistics for visual functioning scale and socioemotional scale are shown in Table 4. Both scales showed a satisfactory fit to the Rasch model with acceptable person separation indices (≥ 2.0), and person separation reliability (≥0.8). Principle component analysis revealed that the first contrast of the unexplained variance had an Eigen value less than 3 suggesting unidimentionality. [19]

**Table 4:** Rasch analysis fit statistics for visual functioning scale and socioemotional scale for NEI-VFQ 25 questionnaire.

Scales	Items in scales	Misfitting items	Person separation index	Person separation reliability	Mean± SD Person measure (logits)	Eigen value
**Visual** **Functioning**	8	2	2.42	0.85	-0.36± 1.49	1.88
**Socio-emotional**	10	2	2.65	0.88	0.18±1.54	2.24

Table 5 reports age and gender adjusted analysis and multivariate analysis of variables affecting the Rasch-calibrated NEI VFQ-25 visual functioning scale and socioemontional functioning scale. Age and gender adjusted analysis (Model 1) showed that worse high contrast VA (p<0.001), higher severity of central VF loss (p = 0.012), and decreased CS (p = 0.001) was assoicated with the low visual functioning subscale in VRQoL. In addition, worse high contrast VA (p = 0.002), higher severity of central VF loss (p = 0.014), and lower CS (p = 0.015) were factors also assoicated with lower socioemontional functioning of VRQoL. Treatment history with anti-vascular endothelial growth factor (VEGF) agents was not associated with VRQoL. A multivariate linear regression model (Model 2) was used, adjusting for possible factors (p<0.10) affecting the patient reported visual function identified in the age and gender-adjusted analysis. High contrast VA (β = -0.25, 95% CI -0.37 to -0.12, p<0.001) and CS (β = 0.35, 95% CI 0.25 to 0.46, p<0.001) were factors associated with the Rasch-calibrated NEI-VFQ 25 visual function scale. High contrast VA (β = -0.2, 95% CI -0.37 to -0.03, p = 0.023) and CS (β = 0.3, 95% CI 0.18 to 0.43, p = 0.001) were also factors assoicated with Rasch-calibrated NEI-VFQ 25 socioemotional function scale. Central VF loss was not assoicated with VRQoL (β = -0.08, 95% CI-0.28 to 0.12, p = 0.44 for visual function scale and β = -0.09, 95% CI -0.03 to 0.16, p = 0.50 for socioemotional function scale).

**Table 5:** Coefficients from regression analyses on factors affecting the vision-related quality of life (VRQoL) using Rasch-calibrated NEI-VFQ 25 questionnaire. Visual function scale included items of general vision, distance vision, and near vision-related acitivity, while socioemotional function scale included items of vision-related social functioning, role difficulties, dependency, and mental health.

	Model 1		Model 2		
	Beta coefficient (95% CI)	P-value	Beta coefficient (95% CI)	Standardized Beta coefficient (95% CI)	P-value
Visual function					
Age (years)	*-0.00041 (-0.005~ 0.004)	0.87			
Gender (Male vs. female)	**0.07 (-0.55~0.69)	0.83			
High contrast VA	-0.76 (-1.16~-0.37)	<0.001	-0.46 (-0.72~-0.21)	-0.25 (-0.37~ -0.12)	<0.001
Severity of central VF loss	-0.43 (-0.76~-0.09)	0.012	-0.11 (-0.39~0.17)	-0.08 (-0.28~0.12)	0.44
CS (log MAR)	1.46 (0.64~2.29)	0.001	1.31 (0.75~1.87)	0.35 (0.25~0.46)	<0.001
Co-morbidities (Yes vs.no)	0.04 (-0.57~0.66)	0.89			
Charles Bonnet (Yes vs. no)	-0.32 (-0.92~0.27)	0.29			
Anti-VEGF treatment (Yes vs. no)	0.40 (-0.1~0.90)	0.11			
Socioemotional function					
Age (years)	*0.00003 (-0.005~0.005)	0.99			
Gender (Male vs. female)	**-0.02 (-0.61~0.58)	0.96			
High contrast VA	-0.70 (-1.14~-0.27)	0.002	-0.36 (-0.67~-0.05)	-0.2 (-0.37~-0.03)	0.023
Severity of central VF loss	-0.40 (-0.72~-0.08)	0.014	-0.11 (-0.44~0.21)	-0.09 (-0.33~0.16)	0.50
CS (log MAR)	1.01 (0.2~1.83)	0.015	1.06 (0.44~1.68)	0.3 (0.18~0.43)	0.001
Co-morbidities (Yes vs.no)	-0.09 (-0.76~0.57)	0.79			
Charles Bonnet (Yes vs. no)	-0.49 (-1.07~0.08)	0.09			
Anti-VEGF treatment (Yes vs. no)	0.41 (-0.13~0.95)	0.14			

Model 1 was adjusted for age and gender except

*adjusted for gender, and

**adjusted for age

Model 2 was adjusted by variables with p value <0.1 in model 1.

VA, visual acuity; VEGF, vascular endothelial growth factor; VF, visual field; CS, contrast sensitivity

VA was categorized according to VA of each eye.; VA of both eyes better than Log MAR VA of 0.48 (Snellen equivalent 20/60), one eye better than 0.48 log MAR, and both eyes worse than 0.48 log MAR.

Estimation of binocular summation of central VF loss was stratified into 13 categories based on presence, location and size of the scotoma. The scoring was then classified into 3 levels of central VF loss severity with mild, moderate and severe VF defect. Presence of co-morbidities such as cardiovascular disease, diabetes mellitus, malignancy or transient ischemic attack was assessed.

## Discussion

Central vision is severely affected secondary to geographic atrophy, disciform scar, or choroidal neovascularization in patients with advanced AMD. Previous studies have used the NEI VFQ-25 to confirm that AMD reduces VRQoL, especially with activities related to central vision, such as reading, driving and facial recognition.[20–23] Therefore, we investigated which clinical factors are significantly associated with VRQoL amongst various factors including decrease in VA, extent of central VF deficit, decrease in CS, age, gender, and other co-morbidities using Rasch-calibrated NEI-VFQ 25 scores. The original NEI VFQ-25 12 –subscale structure was replaced by a two-factor model of visual-functioning scale and socioemotional scale fitting the Rasch model. [24] We confirmed through this study that both scales showed a satisfactory fit to the Rasch model with acceptable separation indices and reliabilities. We determined that the two important factors associated with decreased VRQoL in bilateral advanced AMD patients were lower high contrast VA and lower CS. CS had a stronger association with VRQoL compared to high contrast VA, as the higher absolute value of the standard beta coefficient was higher for CS than for high contrast VA.

Given that monocular VA testing is the most widely accepted method of measuring VA, most studies have focused on the eye with better vision in regards to the VRQoL or categorized eyes into ‘best’ and ‘worst’ eye for analysis. [21, 22] In this study, however, we took into account of VA in both eyes. In the 47 patients included in our study, 23 patients (48.9%) had VA worse than 0.48 LogMAR in both eyes—qualifying them as having ‘moderate vision impairment’ according to the WHO criteria [9]. The remaining patients had a at least one eye with VA better then 0.48 LogMAR, showing the diverse range of VA with advanced AMD. Another factor that we also considered was central VF loss/scotoma secondary to AMD. Macular perimetry determines the presence of scotoma at selected retinal points in patients with unstable fixation who cannot perform traditional VF testing reliably.[25, 26] With SLO-microperimetry, changes in fixation patterns and central retinal sensitivities could be helpful in assessing treatment benefit, visual outcome, and macular function.[27, 28] Midena et al.[25] reported that patients with AMD secondary to subfoveal CNV have a progressive deterioration of VA, along with deterioration of retinal fixation and macular sensitivities on SLO-microperimetry. They suggested that the dense scotoma itself, functionally, can be more limiting to daily-life activities than the loss of VA. Here, we were able to analyze the location of central scotoma secondary to AMD with a novel binocular summation scoring system, accounting for the extent of scotoma in each eye, that categorized the severity of central VF loss in patients with AMD. While age and gender-adjusted analysis showed that worse central VF loss was associated with lower VRQoL on the visual function scale, multivariate analysis revealed that central VF loss loses its impact on VRQoL (p = 0.44). This phenomenon is most likely secondary to high colinearity between the extent of central VF loss and VA (Spearman correlation, ρ = 0.522, P<0.001), possibly due to the small sample size in this study. Although not directly correlated with VRQoL, evaluation of central VF loss using SLO-microperimetry could be an alternative indicator to assess disease progression in patients with severe vision loss secondary to AMD.

Another clinical factor impacting VRQoL for both visual functioning and socioemotional scale is CS. This also has been also shown in previous studies [20][29, 30], where CS was associated with a lower mean score of NEI-VFQ 25 in subcategories of near vision, distance vision, and dependency scores in patients with AMD. Bansback et al. [31] found a relationship between CS and health-related quality of life and health utility, suggesting that benefits of ocular treatments may be underestimated if CS is not considered. Although Maynard et al.[32] suggested that Pelli-Robson CS measured in a mesopic illumination may detect functional deficits earlier than VA testing or VF testing using microperimetry in early or intermediate AMD, CS measured under standard illumination may be sufficient enough to detect functional deficits in patients advanced AMD.

One of the major limiting factors of this study was the number of patients included in this study. Given its retrospective nature and strict inclusion criteria, there were a significant number of patients excluded from this study. Additionally, only central VF loss was assessed, as microperimetry does not have the ability to determine the extent of peripheral VF loss. While one question (#10) in the NEI-VFQ 25 questionnaire asked about peripheral vision loss, the question was reformatted into a visual functioning scale and socioemotional function scale using the Rasch analysis to assess VRQoL. Also, we excluded patients with possible peripheral vision loss (concomitant glaucoma, VF loss caused by stroke) from our study and subsequent analyses. Finally, this study was limited to patients with AMD; therefore, we do not know if these results could be generalized to other diseases that cause central VF loss. Nevertheless, this study has a strength that it evaluated a wide range of clinical factors such as high contrast VA, severity of central VF loss using SLO macular microperimetry, CS, treatment status for AMD, and co-morbidities affecting the quality of life, focusing on patients with bilateral advanced AMD and investigating the association between these factors and VRQoL using the Rasch calibrated NEI VFQ questionnaire.

In conclusion, our study showed that in a series of patients with bilateral advanced AMD, high contrast VA and CS are two factors associated with VRQoL in the NEI VFQ visual function scale and socioemontional function scale.

## Supporting information

S1 File Baseline raw data including baseline demographic data and NEI VFQ 25 questionnaire response. (XLSX)
